# Single-Input and Multiple-Output Surface Acoustic Wave Sensing for Damage Quantification in Piezoelectric Sensors

**DOI:** 10.3390/s18072017

**Published:** 2018-06-22

**Authors:** Lavish Pamwani, Anowarul Habib, Frank Melandsø, Balpreet Singh Ahluwalia, Amit Shelke

**Affiliations:** 1 Department of Civil Engineering, Indian Institute of Technology Guwahati, Assam 781039, India; lavish.p@gmail.com (L.P.); amitsh.iitk@gmail.com (A.S.); 2 Department of Physics and Technology, UiT The Artic University of Norway, 9037 Tromsø, Norway; frank.melandso@uit.no (F.M.); balpreet.singh.ahluwalia@uit.no (B.S.A.)

**Keywords:** surface acoustic wave, empirical mode decomposition, principal component analysis, condition indicator, interdigital transducer

## Abstract

The main aim of the paper is damage detection at the microscale in the anisotropic piezoelectric sensors using surface acoustic waves (SAWs). A novel technique based on the single input and multiple output of Rayleigh waves is proposed to detect the microscale cracks/flaws in the sensor. A convex-shaped interdigital transducer is fabricated for excitation of divergent SAWs in the sensor. An angularly shaped interdigital transducer (IDT) is fabricated at 0 degrees and ±20 degrees for sensing the convex shape evolution of SAWs. A precalibrated damage was introduced in the piezoelectric sensor material using a micro-indenter in the direction perpendicular to the pointing direction of the SAW. Damage detection algorithms based on empirical mode decomposition (EMD) and principal component analysis (PCA) are implemented to quantify the evolution of damage in piezoelectric sensor material. The evolution of the damage was quantified using a proposed condition indicator (CI) based on normalized Euclidean norm of the change in principal angles, corresponding to pristine and damaged states. The CI indicator provides a robust and accurate metric for detection and quantification of damage.

## 1. Introduction

### 1.1. Single Input and Multiple Output

Single Input and Multiple Output (SIMO) and Multiple Input and Multiple Output (MIMO) devices are mainly used in antenna technology for wireless communications [1]. There is growing interest to implement SIMO technology along with orthogonal frequency division multiplexing for high-data-rate underwater acoustic communication [2]. In SIMO, the multiple received signals are able to provide the diversity gain by coherently combining all the signals of interest arriving from a single source [3]. The SIMO and MIMO techniques are used for estimation of the direction of arrival and localization of unknown sources [2,4,5,6]. 

The similar concept of SIMO and MIMO holds a promising feature in nondestructive evaluation (NDE) using ultrasonic waves for identification and quantification of microscale damage in piezoelectric sensors [7]. Ultrasonic guided waves and Rayleigh waves have been extensively used for NDE applications such as mechanical characterization of the thin-film coating, delamination and debonding, and crack detection [8,9,10,11,12,13,14,15]. Continuous inspections of the structure are necessary for ensuring timely maintenance and prevention of catastrophic failure [16,17].

### 1.2. Different Types of Transducers

In the last few decades, a significant number of nondestructive evaluation (NDE) and structural health monitoring (SHM) techniques have been implemented for detecting the failure in critical structures [11,18,19,20,21]. Guided waves such as Lamb waves, Love waves, and SAWs have been widely used for local damage identification in structures [19,22,23,24]. Several experimental techniques were implemented to identify and visualize defects/flaws using acoustic waves, such as ultrasonic probes coupled with angle-adjustable Perspex wedges [25,26,27] and Hertzian contact transducers [28]. The noncontact ultrasonic transducers, such as air-coupled [29,30] and fluid-coupled [31] transducers, and electromagnetic acoustic transducers (EMATs), are other possibilities to generate longitudinal waves [9,32]. However, such transducers have low precision as they suffer from mechanical impedance mismatch between the coupling fluid and the material. Another noncontact excitation and detection technique is based on a pulse laser interferometer that has high precision for wave imaging [33,34,35]. Most recently, the scanning laser vibrometer has been employed for three-dimensional visualization of acoustic wave interference with inclusions and damages in the metallic plates, piezoceramics, piezocrystal and composite plates [36,37,38,39] or in plate-like structures [40]. Rabe and Arnold (1994) have developed acoustic microscopy using atomic force microscopy for characterizing the surface detection in the piezoelectric sensors [41]. However, these techniques are time consuming, expensive and not suited for rapid detection of damage. Lead zirconate titanate (PZT) transducers deliver excellent performance in excitation and detection of Lamb waves and have been employed for damage/delamination detection [42,43,44,45]. The PZT-based excitation of guided waves is limited to low-frequency actuation (typically <1 MHz). PZT usually exhibits certain nonlinear and hysteresis behavior under large strains/voltages or at high temperatures. 

The generation and detection of bulk acoustic waves and Rayleigh waves in piezoelectric crystals with the aid of an interdigital transducer (IDT) has attracted widespread scientific interest for signal processing and filtering applications [46,47,48]. It was first introduced by Mortley [49], for transduction and reception of bulk acoustic waves traveling through the volume of a crystal. The main advantages of IDT over PZT ceramics are: mode selectivity, high excitation strength, wave directivity, small footprint, high excitation frequency and relatively low cost [50,51,52,53]. Interdigital transducers (IDTs) are typically used for excitation and detection of the surface acoustic wave in piezoelectric crystals in the frequency range of 1–125 MHz [54,55]. IDT sensors are also used as strain gauge sensors and guided wave sensors for evaluation of health and quantification of damage in the critical infrastructure. Stoney et al., 2014 [56], Humphries et al., (2015) [57], and Hara et al., (2012) [58] have all developed highly sensitive strain sensors using an SAW resonator for detection of damage in metallic structures of aircraft carriers [59]. The IDT technique has been used to generate Lamb waves in polyvinylidene fluoride piezoelectric polymer films (PVDFs) used for characterization of biological cells, polymers and soft flexible electronics due to its low impedance. However, PVDF polymer transducers have limited frequency bandwidth and low coupling with metallic structures due to impedance mismatch [60]. 

The IDT-based SAW sensors are assumed to be inherently healthy and occasionally calibrated to compensate for error and noise arising from the harsh environmental conditions and temperature fluctuations. For global damage detection, we realistically assumed that the IDT sensor and structural system are inherently coupled. Harsh environmental conditions and extreme loadings such as fatigue, corrosion and high temperature induce damage in the sensors and the structures. Sensors, being sensitive, active devices, usually suffer more damage compared to the structure, and hence acquire an ambiguous response. However, both in research and practice, limited considerations are devoted to quantifying the health of the sensors. The error induced due to the degraded sensor is often circumvented by correcting the acquired response by a baseline compensation factor. Instead of ignoring the performance deterioration of the sensors, we focused our efforts to detect the damage in the sensors. 

The main problem of a conventional IDT sensor is the generation of highly directional and plane-parallel harmonic acoustic waves. The receiver strength of the SAW IDT placed in an angular configuration suffers from losses due to dispersion and dissipation of energy along the thickness of the media. Therefore, the evolution of surface flaw and defects in the off-center path of the conventional IDT will remain undetected due to the low energy content of the diffracted wave. The limitation of low detectability due to scattering and dispersion of the wave is circumvented by fabrication of convex-shaped IDTs. Due to the convex geometrical shape of the IDT, the harmonic waves become divergent and cover a maximum area with uniform energy content along the angular direction. Previously, NDE evaluation of IDT sensors using an angularly placed SIMO with parallel IDT receiver has been demonstrated [7]. 

The above-mentioned experimental techniques aim towards enhancing the detectability of the damage. Most often, the damage is quantified by damage-sensitive features derived empirically using statistical signal processing [19,61]. It is difficult to relate the statistically derived damage index with the physical parameters derived from the theory of wave propagation. In the absence of a theoretical framework, the damage-sensitive features become problem specific and irrelevant for practical application. 

### 1.3. Data Analysis and Evaluation Procedures

The raw data acquired by the IDT sensors behaves as a cryptic signal which does not provide direct measure of damage. Hence, it is extremely important to extract and evaluate sensitive features from the signals for quantification of damage. The selection of appropriate and sensitive features is determined by the material under investigation, and the magnitude and type of damage. The commonly used features are change in the resonating frequency, change in the peak amplitude, reduction in the energy content of the signal, and change in the modal parameters such as mode shape and the eigenfrequencies of the signals. The statistical damage-sensitive features in terms of statistical distance measures are: Mahalanobis distance [62], Mahalanobis distance between phase space topology, and change in phase space topology [63]. The time-series analysis methods such as Auto-Regressive and Auto-Regressive with eXogeneous input models are popular methodologies for damage detection. The time-series methods require data from healthy structures only during the training phase, and this is the main advantage of these methods. The efficient system identification (SI) techniques utilize features derived from wavelet transform, Hilbert–Huang transform, empirical mode decomposition (EMD), principal component analysis (PCA) and so on. SI is challenging in a noisy environment and also for systems possessing low energy. SI techniques work on the assumption that the effect of damage on any system is linear, which is not the actual case. The above-mentioned techniques are mostly adopted on vibration data of the structure to monitor its health. Here, we apply these techniques on ultrasonic wave propagation data to monitor health of a piezoelectric crystal. The algorithm proposed in the current work is a combination of EMD and PCA. The process of EMD gives the dominant intrinsic mode function that has no noise and redundant information. This step helps to avoid false alarm, as any redundant information, if present, will alter the eigenstructure of the signal even in the case of no damage. Later, with the help of PCA, the change in the eigenstructure is evaluated and a sensitive condition indicator is proposed. The condition indicator successfully identifies and quantifies the damage present. 

The novelty of the proposed work is the demonstration of the SIMO technique using excitation with a convex IDT for identification of macroscale damage in piezoelectric sensors. Employing SIMO technology as acoustics sensors in structural health monitoring (SHM) will enhance the detectability and rapid quantification of the damage in the piezoelectric crystal. Also, a new algorithm is proposed using EMD and PCA techniques for signal decomposition and extraction of dominant features to effectively and accurately quantify the damage in the piezoelectric crystal. 

## 2. Interdigital Transducer Fabrication

Interdigital transducers were fabricated on the LiNbO_3_ single crystal. This crystal was cleaned using acetone, isopropanol and trichloroethylene for 15 min at a temperature of 70 °C. After that, the wafer was cleaned using ultrasonic cleaning treatment for 10 min employing deionized water. For removing the moisture content from the wafer, it was baked at 120 °C for 10 min. Later on, the wafer was cooled at room temperature, and positive photoresist (SPR700) was applied to the surface using spin-coating technique at 4000 rpm for 40 s. Later, the wafer was soft baked for 1 min at about 90 °C. After soft-baking, the wafer was exposed to UV light (15 mW/cm^2^) for 10 s. Later on, the sample was baked at 115 °C for 90 s in order to harden the photoresistor. The exposed area of the resistor becomes soluble to the developer (MF26A, Austin, TX, USA).

**Figure 1:**
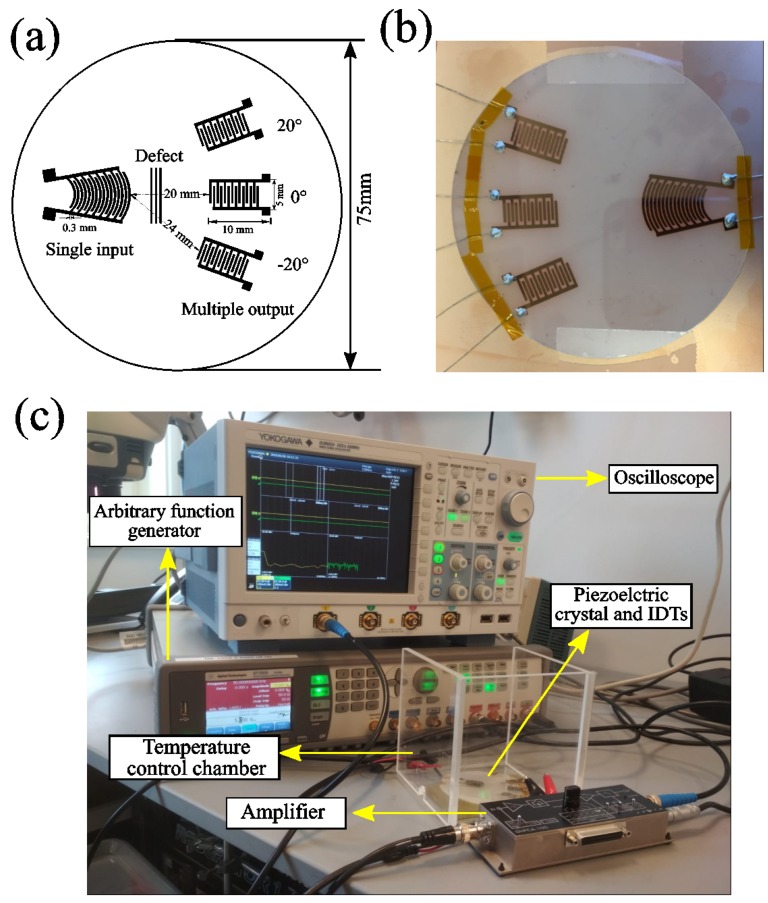
(**a**) Schematic sketch of the Single Input and Multiple Output (SIMO) Inter Digital Transducers (IDT). Convex IDT, marked as single input on the left, three artificial surface defects in the middle of the single input and multiple output has been placed. Signals was received with angularly placed IDTs at 0°, −20°, and 20°, respectively; (**b**) is the optical image of the fabricated sensor; (**c**) is the experimental setup picture.

This developing procedure continued until the section that was exposed to UV light was completely etched (Sigma Aldrich 651818, Darmstadt, Germany). The wafer was sputtered with 0.02 µm of copper using the thermal sputtering technique. The fabricated IDT sensors were connected using conductive epoxy. The schematic diagram of the IDT is shown in Figure 1.

## 3. Experimental Setup and Surface Flaw Generation

**Figure 2:**
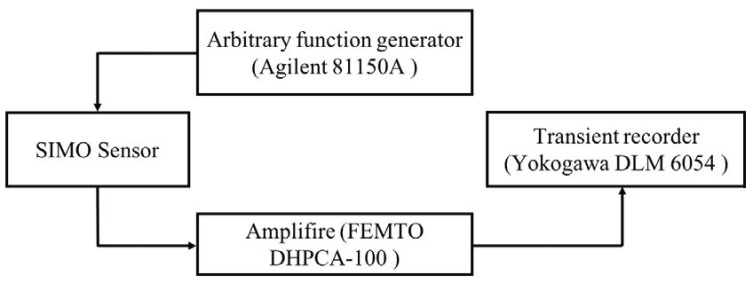
A schematic diagram of the experimental setup of the SIMO IDTs using acoustic waves.

SIMO IDTs were fabricated on the LiNbO_3_ single crystal (Figure 2). The Lamb waves were excited using a curved single-input IDT, and detected signals were received using multiple receivers that were fabricated as a SAW sensor on the LiNbO_3_ crystal. The interspacing distance between two electrodes and the width of the electrode were kept constant. 

The multiple receiver electrodes were placed at angles of 0°, 20° and –20° with respect to the sender electrode. Ideally, it is rather challenging to generate any controlled surface cracks/flaws on a piezoelectric crystal. Therefore, to mimic a surface defect to hinder the Lamb waves’ propagation on the sample, conducting silver paint was spin-coated in between the sender and the receiver IDTs. Using a spin-coater, thickness variation within the preferred area can be controlled by adjusting the spin speed, time, and viscosity of the liquid. Variations in material properties such as surface and bulk defects lead to wave scattering, magnitude-dependent conversion of energy from fundamental to harmonic frequencies in a nonlinear medium, or losses due to viscous contaminants on the surface [64]. The size of the deposit is much bigger than the wavelength of the monitored acoustic waves. The viscous properties of the binding component of the silver paint induce attenuation of acoustic waves, which are additionally strongly scattered by the silver flakes exhibiting a large acoustic mismatch with the binding component. In this way, the deposit from conductive silver paint acts as attenuating and scattering media for acoustic waves at the selected frequency [65,66].

**Figure 3:**
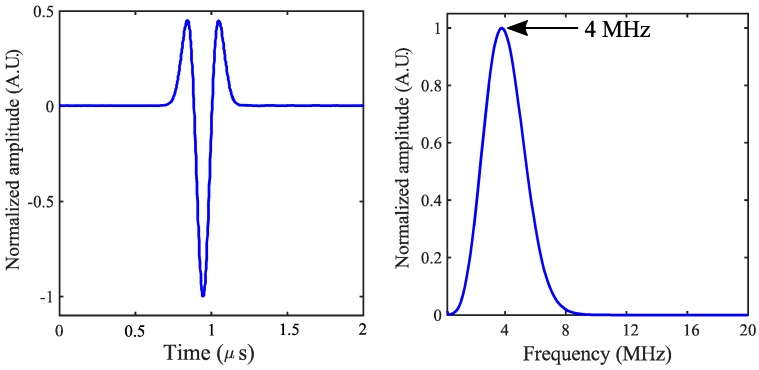
The excitation signal using a broad-banded Gaussian 2nd derivative pulse and; frequency response of the excitation pulse.

Dimensions of the surface flaws are 25 × 3 × 0.75 mm^3^ for the first surface flaws. For the second damage state/flaws, a similar dimension of silver paint was spin-coated on the exactly opposite side of the first flaws. In order to observe the effect of the further defect, a third damage state with a similar dimension has been implemented just beside the first flaws. After inserting every damage state, data were recorded for all the IDT sensors. The received signals from the reference state were compared with that of the damaged state. An arbitrary signal generator (Agilent 81150A, Studio City, California, USA) was used to excite the electrodes with a broad-banded pulse (second derivative of a Gaussian). The signal used for excitation and its frequency content are shown in Figure 3. All the experiments were performed in a thermally insulated chamber to avoid the temperature fluctuation during the experiments.

**Figure 4:**
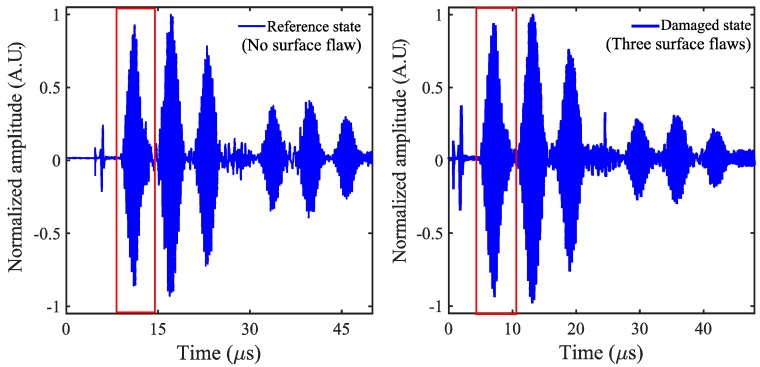
The signal received by IDT sensor placed at 0° orientation corresponding to reference and a damage case (here the damage case considered is corresponding to three surface flaws).

The current induced on a counter-side electrode was then amplified by a transimpedance amplifier (FEMTO DHPCA-100, FEMTO^®^ Messtechnik GmbH, Berlin, Germany). This amplifier converts the current into a voltage according to an adjustable amplification factor, which in the final signal chain, is digitized by the oscilloscope (Yokogawa DLM 6054, Yokogawa Test and Measurement corporation, Mitaka, Tokyo, Japan) after averaging over 256 pulse shootings. For all the measurements, the output of the signal generator was adjusted to provide 5 volts peak to peak, which turned out to be sufficient for producing a good signal-to-noise ratio after averaging over 256 pulse shootings. For the reference and for the damaged state, guided Lamb waves were generated by a single curved sender, and at the same time, signals were received with several interdigital transducers angularly placed. The signal was received by the IDT sensor placed at 0° corresponding to the reference, and a damaged case is shown in Figure 4.

**Figure 5:**
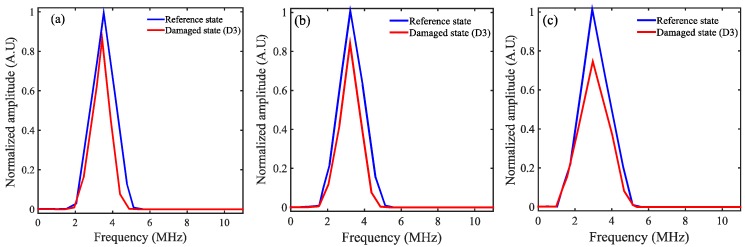
Frequency content of the windowed signal for reference and a damage case corresponding to IDT sensors mounted at (**a**) 0° orientation; (**b**) 20° orientation and (**c**) −20° orientation.

The frequency content of the first wave packet received by IDT sensors is shown in Figure 5. The wave packets considered here are highlighted by the rectangular window in Figure 4. The damage introduced in the LiNbO_3_ crystal will alter the frequency content of the received signal. The change in frequency content can be a potential indicator of damage. In the present case, due to damage, there is no significant change in the frequency content of the received signal, as shown in Table 1. Therefore, there arises a need for a potent and robust condition indicator. In the present work, based on principal component analysis and empirical mode decomposition, a robust damage detection algorithm is proposed. The following sections explain briefly the building blocks of the proposed algorithm.

## 4. Algorithm for Damage Detection 

### 4.1. Principal Component Analysis

Principal component analysis (PCA) is a statistical method that finds combinations of variables or factors that describe major trends in data. It is closely related to proper orthogonal decomposition [67] and singular value decomposition [68]. The main advantage of this method is to reduce the dimensions of multivariate data and retain important information. The overall variation in all the PCs and the original data set is the same [69].

In batch-wise PCA, the data matrix $X\;\in {\;R}^{n\;x\;m}$ is initially modified to a zero-mean matrix $\bar{X}\;\in {\;R}^{n\;x\;m}$ , where $\bar{X}\;=\;X\;-\;E(X)$, *n* is a number of samples, *m* is number of variables and E(X) is mean of the data matrix. Once zero mean data is obtained, the covariance matrix C is calculated as:

(1) $${\left[C\right]}_{m\;x\;m}\;=\;\frac{1}{n-1}{\left[\bar{X}\right]}_{m\;x\;n}^{T}{\left[\bar{X}\right]}_{n\;x\;m}^{}\;$$

The covariance matrix is then processed with eigenanalysis to obtain the matrix P where columns of $P=\left[p_{1},p_{2},p_{3}\ldots {\;p}_{m}\right]$ are eigenvectors of C and the diagonal matrix *λ* with eigenvalues of C.

(2) $${\left[C\right]}_{m\;x\;m}{\left[P\right]}_{m\;x\;m}{\;=\;[\lambda ]}_{m\;x\;m}{\left[P\right]}_{m\;x\;m}$$

The eigenvectors corresponding to smaller eigenvalues are least important and are eliminated. Only the first r eigenvectors corresponding to dominant eigenvalues are selected as PCs. The transformed data set Y after projection in a new space will be r-dimensional:

(3) $${\left[Y\right]}_{n\;x\;r}\;=\;[\bar{X}{]}_{n\;x\;m}{\;[P]}_{m\;x\;r}.$$

The transformed data reduces the computational effort without much loss of information. The projection of Y back to the original data set is ${\mathrm{YP}}^{T}$, and as some eigenvectors are eliminated in the process of PCA it introduces a residual error matrix:

(4) $${\left[E\right]}_{n\;x\;m}\;=\;[\bar{X}{]}_{n\;x\;m}{-\;[Y]}_{n\;x\;r}{\left[P\right]}_{r\;x\;m}^{T}.$$

**Figure 6:**
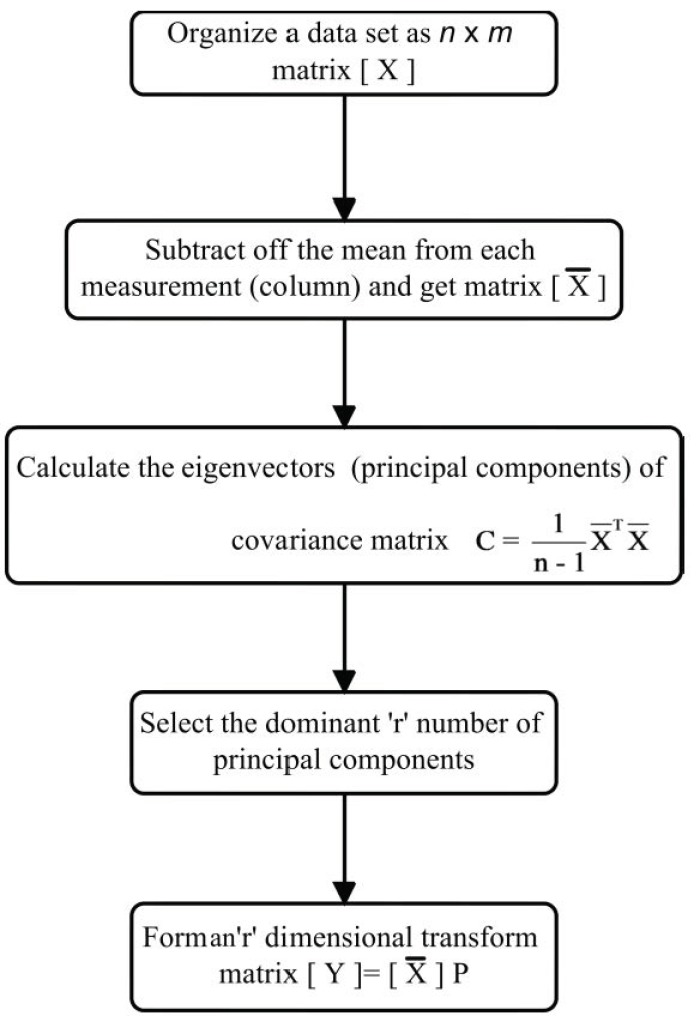
Flowchart representing important steps of principal component analysis.

The flowchart of PCA algorithm is represnted in Figure 6.

### 4.2. Empirical Mode Decomposition

A recorded time history signal x(t) will always contain an inevitable part, referred to as noise. Hence the recorded signal will be an amalgamation of the true signal s(t) and noise n(t)

(5) x(t)=s(t)+n(t)

In the case of nonlinear and nonstationary processes, even though the frequency and time-scale of signal and noise are distinct, it is very difficult to separate noise with the help of conventional filters. Under such circumstances, empirical mode decomposition (EMD) is a useful tool [70]. EMD decomposes a recorded signal into monocomponent signals referred to as intrinsic mode functions (IMFs). The definition of the local mean of two envelopes is quite vague [71]. For a particular ∈, the requirement can be written as:

(6) $${\mathrm{IMF}}_{\in }(t)=\frac{1}{\in }\int_{t-\frac{\in }{2}}^{t+\frac{\in }{2}}\;\mathrm{IMF}(r)\mathrm{dr}=0.$$

An IMF is symmetric, possesses unique local frequency content, and no two IMFs have the same local frequency content at the same time. The evaluation process of IMFs, which satisfies the two important assumptions, is as follows: An IMF candidate for the high-frequency content is determined by foremost fitting a cubic spline over all local minima to construct a lower envelope; an upper envelope is created in an exact fashion. Both the envelopes should cover the whole signal, and their mean is $m_{1}$. The difference between signal and mean is $h_{1}{\;=\;X(t)\;-m}_{1}$. The signal h_1_ is most commonly referred as a proto-mode function (PMF). This PMF has to be processed further to get the true IMF with help of a repetitive processing known as ‘sifting’, where $h_{1}$ is treated as data and sifting is repeated to get:

(7) $$h_{2}={\;h}_{1}{\;-\;m}_{2}.$$

The success of EMD depends on the stopping criteria of sifting. Standard deviation (SD) Equation (8) is one of the ways to stop sifting; as soon as the SD value reaches 0.2–0.3, sifting should stop.

(8) $$\mathrm{SD}=\sum_{t=0}^{T}\left[\frac{|\{{\;h}_{k}({t)\;-\;h}_{k-1}(t)\;\}{|}^{2}}{h^{2}{}_{k}(t)}\right]$$

As soon as the stopping criterion is satisfied, the sifting process is halted; if sifting ends after ‘k’ repetitions, then our first IMF is:

(9) $$c_{1}{\;=\;h}_{k}.$$

This IMF is further subtracted from the signal to obtain a residue $r_{1}{\;=\;X(t)-c}_{1}$. The above procedure of evaluating IMFs is repeated until $r_{n}$ becomes a monotonic function from which no further IMF can be evaluated.

(10) $$\begin{array}{l} r_{1}-c_{2}=r_{2},\\ .\\ .\\ .\\ r_{n-1}-c_{n}=r_{n} \end{array}$$

The final residue may be different than zero, even if the data has a zero mean. To get a signal from IMFs, the following relation works:

(11) $$X(t)=\sum_{i=1}^{n}c_{i}{+r}_{n}.$$

**Figure 7:**
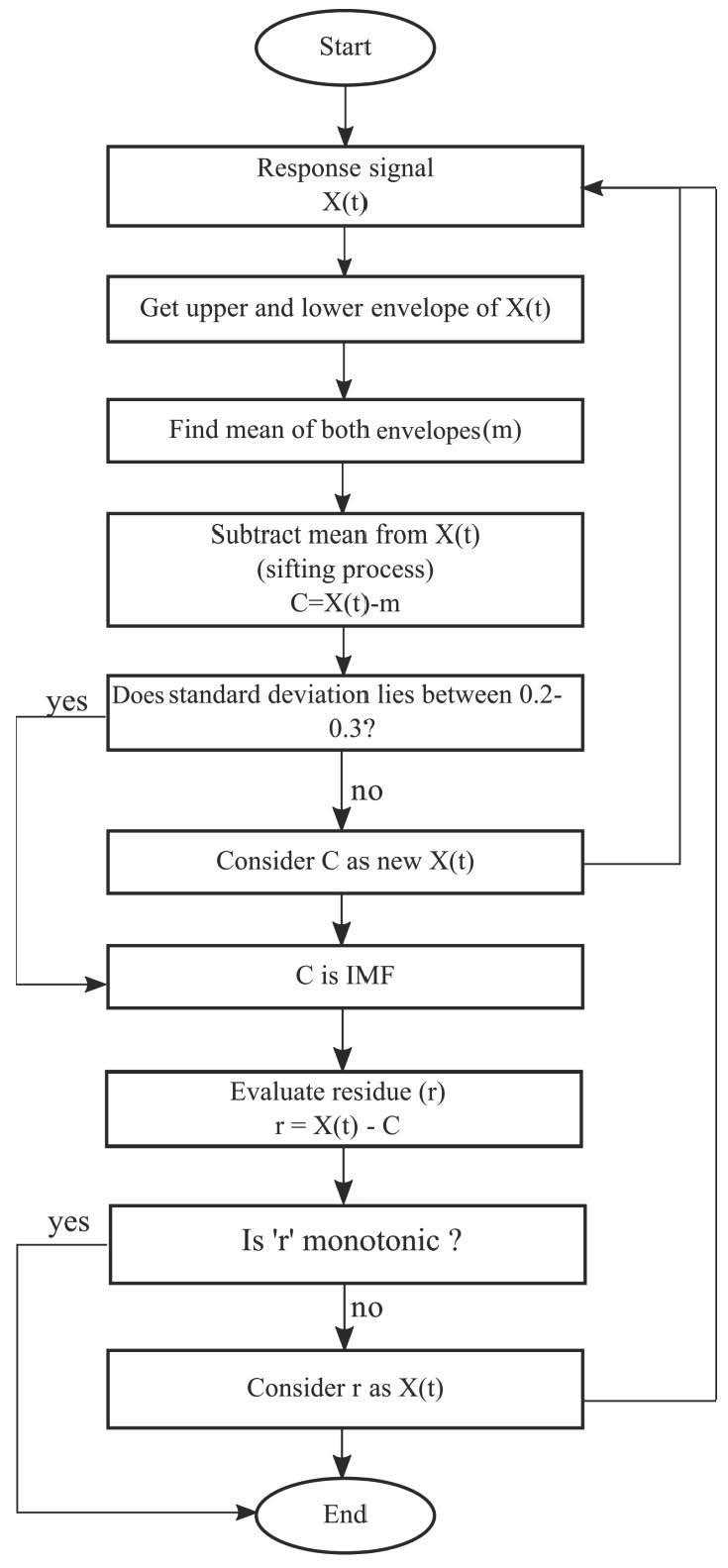
Flowchart representing the sequential steps of empirical mode decomposition.

The entire process of EMD is represented in a flowchart presented in Figure 7.

### 4.3. Algorithm for Defect Detection

(1) Acquire the signal and form a response data matrix
(2) With help of EMD get IMFs corresponding to each signal of response data matrix. Then after, select the dominant IMF for each signal with help of energy and correlation-based approach.
(3) Process all the 2-dimensional combinations of response matrix by PCA and evaluate principal components (PCs) for each. Repeat the above process for baseline as well as damage states.
(4) Evaluate the condition indicator (CI) based on change in the angle of PCs. The detailed explanation of CI is in Section 5.2. The CI successfully segregates damage state from healthy state, it also quantifies the defect.

The above-mentioned algorithm is explained in detail considering the example presented in the current work:

(1) The piezoelectric crystal is excited by a broadband pulse and the response is acquired by three IDT sensors mounted. The acquired data forms a response data matrix [A] of size N × 3, where *N* is total number of samples acquired, and the numeric 3 is due to three IDT sensors.
  $$A={\left[\begin{array}{ccc} x_{1} & y_{1} & z_{1}\\ \vdots & \vdots & \vdots \\ x_{N} & y_{N} & z_{N} \end{array}\right]}_{N\;x\;3}$$
  The EMD of each column vector of matrix A will result in multiple IMFs corresponding to each:
  $$\mathrm{EMD}\left(\begin{array}{c} x_{1}\\ \vdots \\ x_{N} \end{array}\right)=\left[\begin{array}{c} {\{\mathrm{IMF}}_{1x}\}\\ \vdots \\ {\{\mathrm{IMF}}_{\mathrm{mx}}\} \end{array}\right],\;$$
  where *m* is the number of IMFs obtained after EMD and ${\{\mathrm{IMF}}_{1x}\}$ is a row vector.
  The next step is to calculate energy and correlation of each monocomponent IMF. The IMF corresponding to maximum correlation and energy is selected as the dominant IMF. In the present case, ${E\{\mathrm{IMF}}_{1x}{\}<\mathrm{MF}}_{2x}{\}>\mathrm{MF}}_{3x}\}>\cdots {>\mathrm{MF}}_{\mathrm{mx}}\}$, the results for correlation are the same. Therefore, the second IMF is selected as the dominant IMF.
(2) In this step, we form all the possible two-dimensional combinations of the response matrix, and process it by PCA. Considering the response matrix of the current work, the three variables will lead to three two-dimensional combinations:
  $$C-1=\left[\begin{array}{c} x_{1}\\ M\\ x_{N} \end{array}\begin{array}{c} y_{1}\\ M\\ y_{N} \end{array}\right],C-2=\left[\begin{array}{c} y_{1}\\ M\\ y_{N} \end{array}\begin{array}{c} z_{1}\\ M\\ z_{N} \end{array}\right],C-3=\left[\begin{array}{c} x_{1}\\ M\\ x_{N} \end{array}\begin{array}{c} z_{1}\\ M\\ z_{N} \end{array}\right],$$
  where C-1, C-2 and C-3 represent combination one, combination two and combination three, respectively.
  The PCA of a combination will give its principal components and principal angles. The detailed explanation of PCA is given in Section 4.1. $\theta_{d}$ and $\theta_{\mathrm{ref}}$ are principal angles corresponding to a damaged case and healthy (reference) case, respectively.
(3) Using the value of principal angle for reference and damaged cases, we evaluate the condition indicator (CI) proposed in the current work.
  $$\mathrm{CI}=\sqrt{\frac{\sum {{(\theta }_{d}{-\theta }_{\mathrm{ref}})}_{i}^{2}}{\sum {{(\theta }_{\mathrm{ref}})}_{i}^{2}}}$$
  where i is number of combinations of data.

**Figure 8:**
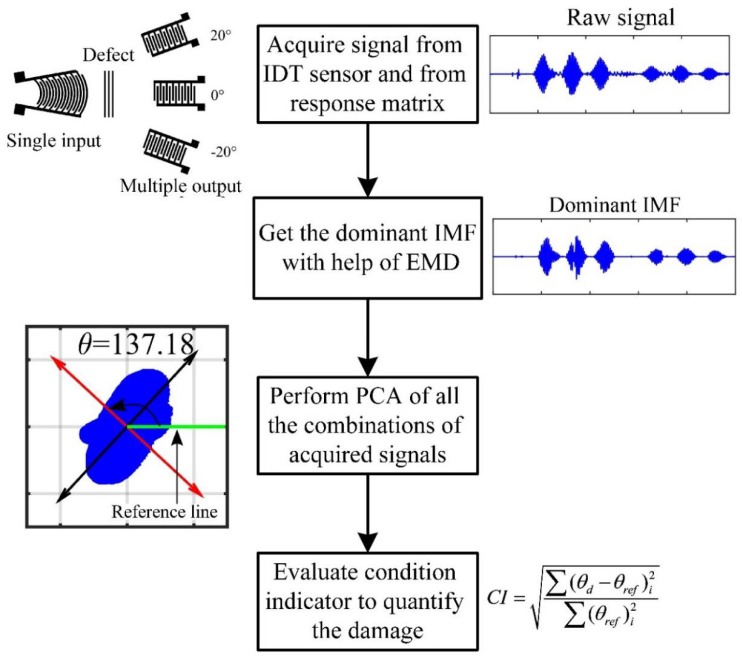
Flowchart of the proposed damage detection algorithm.

In the present case, the value of i = 3, as mentioned in step three. The flowchart of the entire algorithm is presented in the Figure 8.

The minimum dimensions of the response matrix for the proposed algorithm to work is two. This is because the current work is based on PCA. Based on the concept of PCA, the order of the response matrix shall be at least N × M, where N is the length of the response signal and M is the minimum number of discrete sensors. There is no limitation for the maximum dimensions of the response matrix. However, in any SHM framework, our goal is to detect damage and abnormality using the smallest number of sensors. In cases where the dimensions of the response matrix are higher, it will lead to a higher number of combinations for PCA. The large dimension of the response matrix will only increase the computation time. 

## 5. Results and Discussion

The response signals captured by IDT sensors placed angularly on the piezoelectric crystal (Figure 1) are collected for different states, that is, the pristine state and the damaged states. The following section explains the results and conclusions for damage quantification based on the proposed algorithm. Initially, we discuss the selection of the dominant IMFs based on two different approaches. Further, we discuss an approach based on PCA to obtain the principal components. Lastly, a condition indicator is proposed to quantify the damage induced in the LiNbO_3_ sensors. The condition indicator is based on a change in angle between PCs of the healthy and damaged states. 

### 5.1. Empirical Mode Decomposition of Response Signals

**Figure 9:**
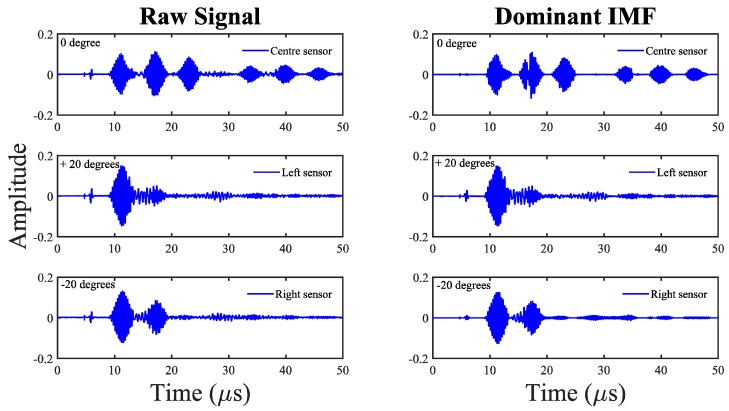
Raw signals and their corresponding dominant IMFs for the baseline state of all IDT sensors.

The response signals from the receiver IDTs are decomposed into their intrinsic mode functions (IMFs) with the help of the EMD technique explained in Section 4.2. Each of the IMFs generated represents an inherent timescale characteristic of the original signal, and these are monocomponent signals. Figure 9 shows the dominant IMFs for pristine state signals received by all the IDT sensors. In order to accurately quantify the damage induced in the piezoelectric crystal, the response signals are analyzed through their dominant IMFs. The dominant IMF is one that has maximum energy and correlation with the original signal; also, it has no vague information (e.g., high-frequency noise). The selection of the dominant IMF is done by two different approaches, namely, an energy-based approach and a correlation-based approach.

#### 5.1.1. Energy-Based Approach

The first approach is the average energy content of the IMF, which can be calculated in discretely sampled signals as:

(12) $${E(x}_{j}(t))=\frac{\sum_{i=1}^{N}{\left|x_{j}{(t}_{i})\right|}^{2}}{T},$$

where $x_{j}(t)$ represents the $j^{\mathrm{th}}$ IMF of the measured signal x(t), T is the total duration of the signal and *N* is the total number of data points. The energy content in an IMF would be directly proportional to its contribution towards the original signal. 

#### 5.1.2. Correlation-Based Approach

The second approach evaluates the correlation between the original signal and each of the IMFs. The IMF that has a maximum correlation with the original signal is selected as the most dominant IMF. The correlation coefficient is calculated as

(13) $${\Lambda (x(t),x}_{j}(t))=\frac{C_{{x(t)x}_{j}(t)}}{\sigma_{x(t)}\sigma_{x_{j}(t)}},$$

where x(t) and $x_{j}(t)$ represent the original signal and its $j^{\mathrm{th}}$ IMF, $C_{x(t)x_{j}(t)}$, is the covariance between x(t) and $x_{j}(t)$, $\sigma_{x(t)}$ and $\sigma_{x_{j}(t)}$ are the standard deviations. 

**Figure 10:**
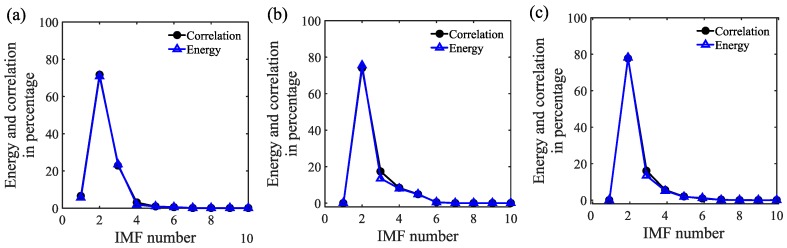
Energy content and correlation of all IMFs corresponding to signal acquired by: (**a**) Centre (0°); (**b**) Left (20°) and (**c**) Right (−20°) IDT sensor for reference state.

The energy and correlation of IMFs calculated using the above procedure are normalized with respect to the maximum value and then represented in percentage, as shown in Figure 10. The most strongly contributing IMFs according to the above two criteria are the ones that possess a maximum percentage of energy and correlation. The dominant IMFs are considered instead of the original response signal for further analysis. This step allows de-noising of the signal and facilitates removal of ambient disturbances.

Once the dominant IMFs are selected for the reference and damaged states, they are used to quantify damage using principal component analysis as explained in the following section. 

### 5.2. Principal Component Analysis of Dominant Intrinsic Mode Functions

The IDT sensors’ angular arrangement for our experiment provides three response signals from the 0° (center sensor), 20° (left sensor) and –20° (right sensor) orientations embedded in the piezoelectric sensor. Assuming X to be the response matrix for a given state of the piezoelectric crystal (i.e., reference or damaged state), the columns of matrix X consist of the response signal received by all the IDT sensors. For PCA, instead of the original response matrix, we form another matrix, X*_IMF_*. The columns of X*_IMF_* consist of the dominant IMFs corresponding to each column of the response matrix X.

In the present case, we have one healthy and three damaged-state response data matrices that lead to four different X*_IMF_* matrices. The damage is induced by a macro surface flaw in the lithium niobate (LiNbO_3_) piezoelectric crystal. The following section provides a quantitative representation of the damaged states with respect to the reference state by means of PCA. PCA is a tool for realizing a given set of data in a transformed set of coordinates which enables reducing inherent redundancy in the data. The transformed coordinates are a combination of the original coordinates, and are uncorrelated with each other. This tool helps in revealing the principal structure of data, which can be exploited as a damage quantification tool. 

Consider the matrix X*_IMF_* of dimensions *n* x *m*, where *n* is the number of observations and *m* is the type of observed data (dimension of PCA); we get the covariance matrix ([C]*_m_*_x *m*_) of X*_IMF_***.** Now, as explained in Section 4.1, the eigenanalysis of the covariance matrix gives the principal components (PCs). Equation (2) represents the eigenanalysis of the covariance matrix ([C]*_m_*_x *m*_), where the columns of [P] are PCs.

**Figure 11:**
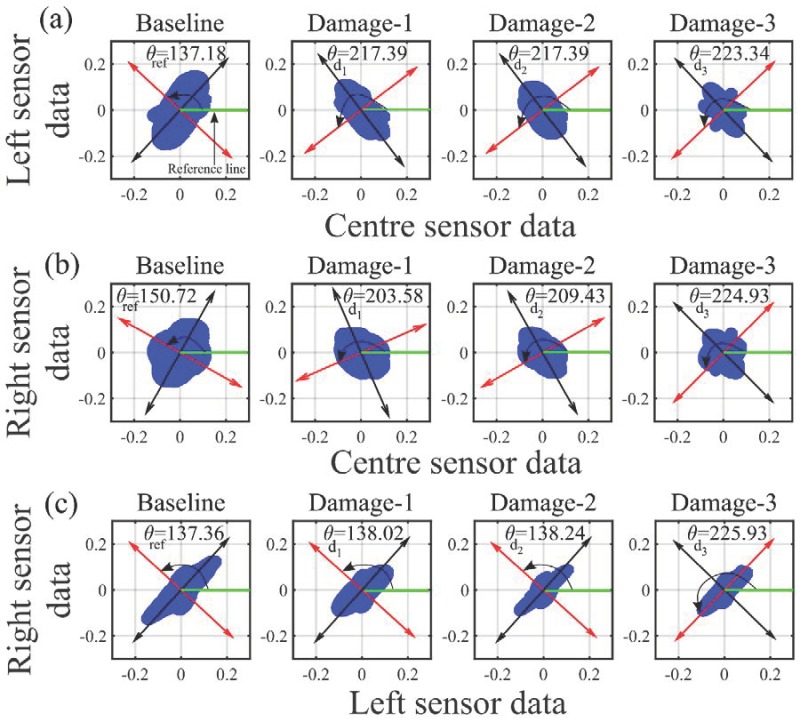
Principal components corresponding to healthy and damaged states for: (a) combination one; (**b**) combination two and (**c**) combination three.

The PCs obtained using PCA are an important indicator of the underlying eigenstructure of the data. In our case, we monitor the changes in the eigenstructure of the matrix X*_IMF_* corresponding to the reference and damaged states. This step is pursued on the reasoning that a change in the state of the piezoelectric crystal will distort the eigenstructure of the response signal. The distortion in the eigenstructure of the data is quantified based on the changes in the angle of the PCs obtained from PCA. Let $\theta_{\mathrm{ref}}{,\theta }_{d_{1}}{,\theta }_{d_{2}}$ and $\theta_{d_{3}}$ be the angles of PCs with respect to the reference axis corresponding to the healthy and three damaged states, respectively (Figure 11).

In the present case, as the total number of IDT sensors receiving signal is three, the total number of combinations for two-dimensional PCA is three, as explained in Section 4.3. The three IDT sensors shown in Figure 1 are represented as left (20°), right (–20°) and centre (0°) sensors. Therefore, the possible three combinations for PCA are combination one: left and centre, combination two: right and centre, and combination three: right and left.

For all three combinations of the measured signal matrix, $\theta_{\mathrm{ref}}{,\theta }_{d_{1}}{,\theta }_{d_{2}}$ and $\theta_{d_{3}}$ are computed as shown in Figure 11. Based on the measurements of $\theta_{\mathrm{ref}}{,\theta }_{d_{1}}{,\theta }_{d_{2}}$ and $\theta_{d_{3}}$, a condition indicator (CI) is proposed as follows:

(14) $$\mathrm{CI}=\sqrt{\frac{\sum {{(\theta }_{d}{-\theta }_{\mathrm{ref}})}_{i}^{2}}{\sum {{(\theta }_{\mathrm{ref}})}_{i}^{2}}}$$

where i is total number of combinations of data matrix

The values for the CI have been tabulated in Table 2. 

From the value of CI, we can clearly see that case damage-3 reflects more severe damage compared to damage-1 and damage-2. This parameter helps us to gauge the relative degree of damage induced in the material for the different damage states of our experimental study.

## 6. Conclusions

The outcome of this paper is to quantify the surface flaws in the anisotropic piezoelectric single crystal (LiNbO_3_) using PCA and EMD. The surface aberration of the sensors is created using parallel surface flaws. The incubation of surface flaws/damage at the macro scale act as a precursor to the macroscale effect, which is an indicator of the evolution of damage. The SIMO technique has been employed for excitation of convex shape evolution of the wave. In this study, the divergent IDT sender generates the convex-shaped wave field in the LiNbO_3_ crystal. For structural health assessment of the piezoelectric sensor, Rayleigh wave signals were acquired at different angles through angularly placed IDTs on the surface of the LiNbO_3_ crystal. In the healthy scenario, the convex-shaped wave field possesses the same intensity at equidistant points from its source, whereas the presence of a defect in the perpendicular direction of wave propagation leads to a significant scattering of the convex wave, resulting in a loss in the amplitude and energy of the signal. Therefore, the waves received by the IDTs are low in magnitude and energy, and this makes it more difficult to fetch information of excited modes and monitor the health of the crystal. The proposed algorithm implements EMD on the received wave and segregates dominant modes as the dominant IMFs. Subsequently, the IMF is processed by the proposed damage detection algorithm to evaluate the condition indicator. The manifestation of damage and its evolution is quantified with help of the change in the value of the condition indicator (CI). The value of the CI corresponding to damaged cases of the present work are 0.39, 0.40 and 0.58. Instead of determining the exact size of the surface flaw, we quantify the magnitude of the damage with the help of CI. The relative change in magnitude of CI reflects the manifestation and severity of damage. The proposed framework is capable of quantifying the damage in the form of surface flaws with help of a condition indicator that could be applicable for various SHM frameworks. 

## Figures and Tables

**Table 1:** Maximum frequency content of the windowed signal for reference and a damage case corresponding to IDT sensors mounted at 0° orientation; 20° orientation and −20° orientation.

	IDT Sensors Fabricated at		
	0° Orientation	20° Orientation	° Orientation
Reference state	3.58 MHz	3.5 MHz	3.46 MHz
Damaged state—3	3.45 MHz	3.42 MHz	3.4 MHz
% change in frequency	3.63	2.2	1.77

**Table 2:** Quantitative changes in Condition indicator for various damage scenarios.

Damage Cases	Damage-1	Damage-2	Damage-3
**CI values**	0.391	0.405	0.587
**% change in CI**	-	3.5	50.12

## List of Notations

x(t)	Acquired signal
$x_{j}(t)$	*j*th IMF of the signal x(t)
*T*	total duration of the signal
*N*	the total number of data points
*C*	Covariance matrix
*E*	Residual error matrix
$C_{{x(t)x}_{j}(t)}$	covariance between ${x(t)\;\mathrm{and}\;x}_{j}(t)$
$\sigma_{x(t)}$	standard deviation of x(t)
$\sigma_{x_{j}(t)}$	standard deviation of x_j_(t)
X	Response matrix
$\bar{X}$	Zero mean response matrix
*X_IMF_*	Matrix consisting of IMFs corresponding to response matrix
LiNbO_3_	Lithium Niobate
$\theta_{\mathrm{ref}}$	Principal angle corresponding to reference case
$\theta_{d_{i}}$	Principal angle corresponding to damage-*i* case
